# Non-alcoholic Fatty Liver Disease Causing Platypnea-Orthodeoxia Syndrome

**DOI:** 10.7759/cureus.11727

**Published:** 2020-11-27

**Authors:** Hassam Ali, Zarafshan Shafique, Alina Sehar, Sreeram Yalamanchili

**Affiliations:** 1 Internal Medicine, East Carolina University, Greenville, USA; 2 Internal Medicine, Quaid-E-Azam Medical College, Bahawalpur, PAK; 3 Internal Medicine, United Medical and Dental College, Karachi, PAK

**Keywords:** nonalcoholic fatty liver disease (nafld), platypnea, orthodeoxia, heart failure, heart failure with preserved ejection fraction, hypoxic respiratory failure, hepatopulmonary syndrome

## Abstract

Non-alcoholic fatty liver disease (NAFLD) with cryptogenic liver cirrhosis is uncommonly linked to platypnea-orthodeoxia syndrome (POS). Traditionally, this syndrome has been described in correlation to intracardiac shunting like patent foramen ovale.

We report a case of a 70-year-old female, with a previous history of NAFLD and heart failure presenting with acute hypoxic respiratory failure secondary to fluid overload. Further investigations revealed cryptogenic presentation of POS, which was masked by her heart failure. The patient was not able to maintain her oxygen saturation levels in an upright position, with marked improvement when lying down. Her echocardiogram was significant for positive bubble study without any intracardiac shunt, hence making NAFLD as a cause of this rare presentation of POS a more likely diagnosis.

## Introduction

Nonalcoholic fatty liver disease (NAFLD) is the presence of hepatic steatosis when no secondary causes of hepatic fat accumulation (for example, alcoholism) are present. NAFLD may progress to cirrhosis and is likely a notable cause of cryptogenic cirrhosis [1]. Factors associated with NAFLD include old age, systemic hypertension, obesity, and diabetes [2]. NAFLD can lead to cirrhosis and thus often associated with intrapulmonary vascular dilations, leading to platypnea-orthodeoxia syndrome (POS). Although POS has been more commonly linked to intracardiac shunts like patent foramen ovale, NAFLD associated POS has been rarely reported in the literature [3].

## Case presentation

**Figure 1:**
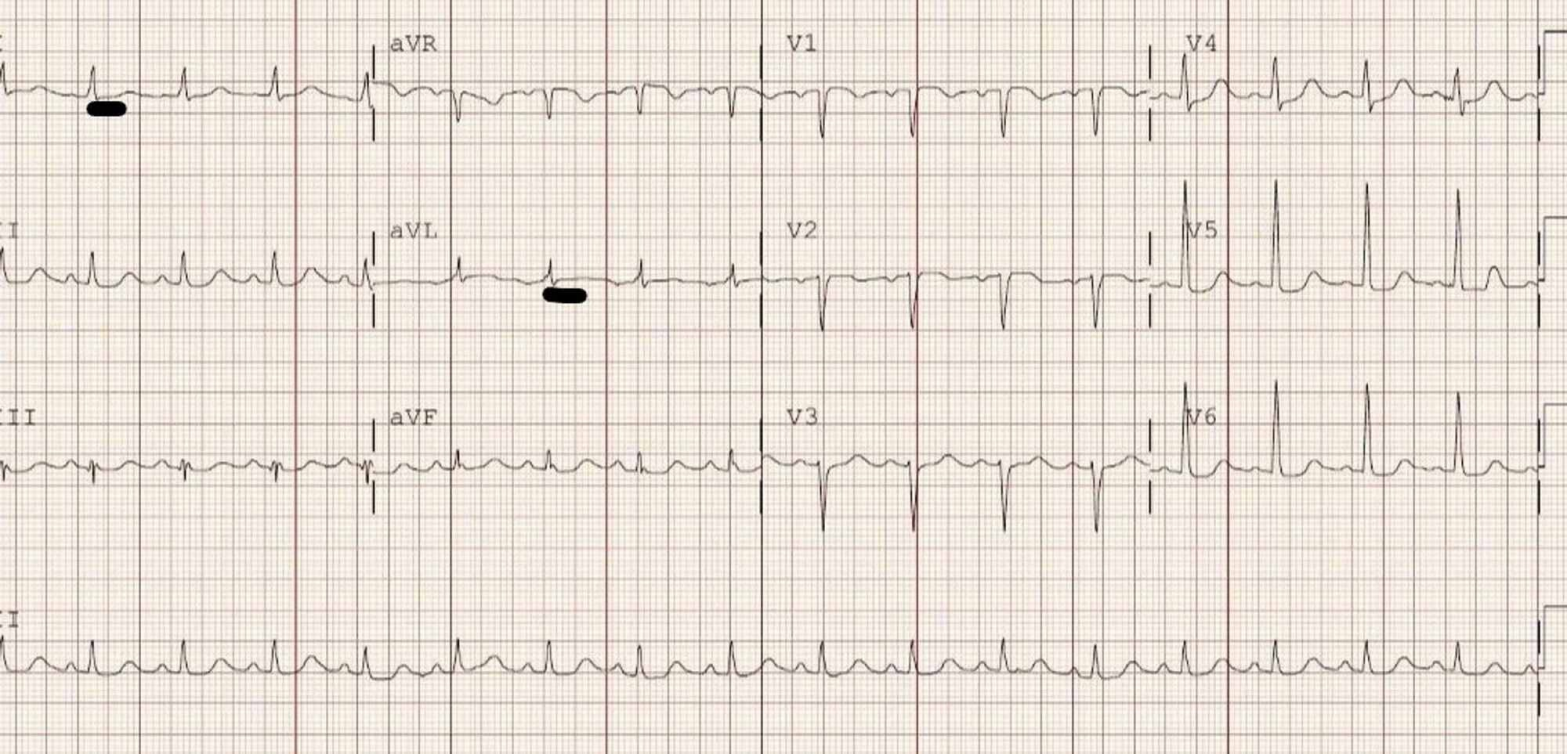
Electrocardiogram showing minimal ST segment depression in leads I and aVL. aVL, augmented Vector Left; aVR, augmented Vector Right; aVF, augmented Vector Foot.

A 70-year-old female presented to the emergency department with acute hypoxic respiratory failure. The patient had a significant previous medical history of pulmonary hypertension with heart failure, hypertension, diabetes mellitus, and NAFLD with liver cirrhosis. She was desaturating with oxygen saturation of less than 80% on room air. Physical examination revealed bilateral rales. Vitals included a blood pressure of 136/75 mmHg, pulse 98 beats/minute, temperature 98°F, and a respiratory rate of 26 breaths/minute. She did not respond well to oxygen visa nasal canula, so bilevel continuous airway pressure (BiPAP) was used. Initial chest x-ray showed bilateral pulmonary edema, and she received a bolus of furosemide that improved her oxygen saturation. She was later weaned down to 4 liters (L)/minute nasal cannula. Further history revealed that the patient had increased dyspnea on exertion and some chest pain with palpitations since last few days. Electrocardiogram (EKG) was significant for ischemic changes in lateral leads (Figure 1). Serum troponin was elevated up to 2.74 ng/dl. Echocardiogram was significant for mildly dilated left ventricle with an ejection fraction of 55%-60% and moderate aortic valvular stenosis with mean gradient 20 mmHg. Due to concerns of non-ST-segment elevation myocardial infarction (NSTEMI), she underwent a left heart cardiac catheterization, which was negative for coronary artery disease. During her hospital course, she continued to have episodes of oxygen desaturation, which were found to have a positional component. Her oxygen levels would drop below 80% when asked to stand up, despite being on 4 liters (L)/minute nasal cannula, but improve to 98% when asked to lay flat. The patient was investigated for POS with concerns of patent foramen ovale. The echocardiogram with bubble study was positive (Video 1), but no intracardiac shunt was identified on right heart catheterization. Computed tomography angiography did not show any large-vessel pulmonary arteriovenous malformations or dilations, and a nuclear medicine pulmonary perfusion imaging was recommended, which was deferred as it would not have changed the prognosis or treatment. Abdominal ultrasound showed coarse hepatic echotexture with diffuse nodularity consistent with cirrhosis. Thus a tentative diagnosis of POS due to her NAFLD was made. Unfortunately, the patient was not a candidate for liver transplant and later discharged with conservative management.

**Video 1:** Positive bubble study during an echocardiogram

## Discussion

POS is characterized by an increase in breathlessness on standing up but gets back to normal on changing from upright position to recumbent position. POS can also be associated with hepatopulmonary syndrome (HPS), which is defined as a triad of liver cirrhosis, insufficient oxygen, and pulmonary vascular dilatation. It is not necessary that all cirrhotic patients with HPS will have POS [4]. It can be a classic manifestation of HPS, but it is not diagnostic. Cirrhosis alone, even without HPS, is capable of altering pulmonary vasculature resulting in platypnea affecting areas of alveoli with insufficient blood perfusion [5]. Among the causes of cirrhosis, NAFLD is among one of the emerging causes of liver cirrhosis associated with little or no alcohol intake. A more severe presentation of NAFLD is non-alcoholic steatohepatitis (NASH), which is defined as steatosis, hepatocyte ballooning, fibrosis, and inflammation of lobules [6]. The presence of POS is either due to intrapulmonary shunt, intracardiac shunt, ventilation-perfusion mismatch, or a combination of these entities [7]. Sometimes, a patent foramen ovale may remain undiagnosed until a late age. So when an elderly patient presents with position-dependent hypoxemia, POS of cardiac origin must be ruled out by carrying out necessary investigations [8].

Non-invasive tests are generally used to diagnose POS. There are two available tests that can define intrapulmonary dilatations. Contrast transthoracic echocardiography, which most commonly uses microbubbles as the contrast, can demonstrate the presence of intrapulmonary shunting, which causes ventilation-perfusion mismatch and ultimately hypoxemia [9]. The use of transthoracic echocardiography (TTE) is preferred over transesophageal echocardiography (TEE) in case of patients with cirrhosis due to the frequent presence of esophageal varices, which can massively bleed on touch. The other test is a radioactive lung perfusion scan using macro-aggregated albumin (MAA). The bubble contrast echocardiography has proven to be more sensitive than MAA, and it is also less invasive, promptly available, and can exclude intracardiac shunting. So it is considered as the method of choice for the screening purposes [9].

For the diagnosis of HPS, evidence of liver disease, hypoxemia, and intrapulmonary shunt (intrapulmonary vascular dilatations or pulmonary arteriovenous malformations) must be present [5]. In our patient, a TTE with bubble study was positive, but right heart catheterization revealed no intracardiac shunt such as patent foramen ovale or a septal defect. Computed tomography angiography (CTA) failed to show significant intrapulmonary dilatations; so the diagnosis of classic hepatopulmonary syndrome was deferred as the triad was incomplete. However POS in the setting of liver cirrhosis and negative workup intracardiac shunting may be a herald for HPS. Unfortunately, HPS is only treatable with liver transplantation; which was not a possibility in our patient.

## Conclusions

POS in the setting of liver cirrhosis and negative workup of intracardiac shunting may be a herald for hepatopulmonary syndrome. It is vital to rule out other causes like intracardiac shunting or pulmonary atriovenous malformations. Early recognization of POS in patients with liver cirrhosis, specially NAFLD, may warrant early liver transplantation before hepatopulmonary syndrome sets in, as the wait time for liver transplant is quite long and HPS has increased mortality in cirrhotic patients. Unfortunately, once progressed to hepatopulmonary syndrome, there is no other alternative to liver transplantation.
